# Supplementary material for: Prevalence and correlates of overweight and obesity among primary school children in Kilimanjaro, Tanzania
Source: PLoS One. 2021 Apr 22;16(4):e0249595. doi: 10.1371/journal.pone.0249595 (PMC8061999; doi:10.1371/journal.pone.0249595)
Supplement: S1 File — (DOCX) [file pone.0249595.s002.docx]

**2019 Physical activity and Diet survey**

**Participant ID: ____ ____ ____ Date** ____ / ____ /

**dd mm yyyy**

**School name:_______________________ School type____________________**

**District:______________________ Ward:_________________**

**Interviewer: _______________________**

Please read every question carefully. What answer comes to your mind first?

Choose the box that fits your answer best and fill it in.

Remember: This is not a test so there are no wrong answers. It is important that you answer all the questions and that we can see your marks clearly.

You do not have to show your answers to anybody. Also, nobody who knows you will look at your questionnaire once you have finished it.

**Socio demographic characteristics**

Date of birth _____/_____/_____ Age (years)__________

dd mm yyyy

Sex

- Male
- Female

Which class are you?

- Standard four
- Standard five
- Standard Six
- Standard Seven

**Section 1a**

For the questions on this page, please tell about what you did *last week*.

**Sedentary habits:**

Most questions will ask you to think only about the LAST 7 DAYS, but a few questions will ask about what you typically do (during a normal week). THERE ARE NO RIGHT OR WRONG ANSWERS SO PROVIDE HONEST ANSWERS.

SEDENTARY HABITS refer to activities such as watching TV, or playing video games, computer games, or browsing the Internet or playing hand-held games. It includes time spent using a phone to talk or text with friends, play games.

On school days, (Monday – Friday)

1. a) How many days last week did you watch television?

- I did not watch
- 1 day
- 2 days
- 3 days
- 4 days
- 5 days

b) How long did you watch television on each day you watched?

- I did not watch
- Less than one hour (1 to 30 minutes)
- One hour (31 – 60 minutes)
- 1 to 2 hours
- More than two hours

1. a) How many days last week did you play video games, play with computer or use computer on non-school activities?

- I did not watch
- 1 day
- 2 days
- 3 days
- 4 days
- 5 days

b) How long did you play video games, play with computer or use computer on non-school activities?

- I did do this
- Less than one hour (1 to 30 minutes)
- One hour (31 – 60 minutes)
- 1 to 2 hours
- More than two hours

1. a) How many days did you spend reading or just sitting, by yourself or with friends or family?

- I did not watch
- 1 day
- 2 days
- 3 days
- 4 days
- 5 days

1. How long did you spend reading or just sitting, by yourself or with friends or family?

- I did do this
- Less than one hour (1 to 30 minutes)
- One hour (31 – 60 minutes)
- 1 to 2 hours
- More than two hours

On weekends, (Saturday and Sunday)

1. How long did you watch television on these days

- I did not do this
- Less than one hour (1 to 30 minutes)
- One hour (31 – 60 minutes)
- 1 to 2 hours
- More than two hours

1. Hong long did you play video games, play with computer or use computer on non-school activities?

- I did not do this
- Less than one hour (1 to 30 minutes)
- One hour (31 – 60 minutes)
- 1 to 2 hours
- More than two hours

1. How long did you spend reading or just sitting, by yourself or with friends or family?

- I did do this
- Less than one hour (1 to 30 minutes)
- One hour (31 – 60 minutes)
- 1 to 2 hours
- More than two hours

**Physical activity**

This section asks about physical activities that is, any play, game, sport, exercise at home or school (either during recess or after school), transportation (like walking or cycling to school) that gets you moving and breathing harder.

1. In a typical week, how many days have you participated in physical education/ exercise (PE) classes, this means practical sessions

- 0 day
- 1 day
- 2 days
- 3 days
- 4 days
- 5 days

1. In a typical week, do you normally walk to school?

- YES
- NO

IF THE ANSWER FOR QUESTION 8 IS NO, SKIP TO QUESTION 9.

1. How much time do you normally walk to reach to school?

- Less than 5 minutes
- 5 – 15 minutes
- 16 – 30 minutes
- 31 minutes to 1 hour
- More than 1 hour

1. On school days, when you wake up in the morning while you are at home do you normally activities such as housework, gardening, fetching water etc before going to school for at least 10 minutes?

- YES
- NO

1. On school days when you are at home after school, do you normally do activities such as housework, gardening, fetching water, sweeping the ground, mopping, and washing etc for at least 10 minutes?

- YES
- NO

1. When you are at school, do you spend some minutes during break time to do some physical activity like playing netball/ football, skipping etc

- YES
- NO

1. Do your parents make you exercise or encourage you to go out to play?

- YES
- NO

**Section 2a**

Food Frequency Questionnaire

This section is asking about how many times (in a typical week) do you usually eat the following foods (Please mark only one box on each line)

|  | Never | Once per day | More than once per day | 3 – 6 times per week | Once or twice per week | Everyday, more than once |
| --- | --- | --- | --- | --- | --- | --- |
| Fruits (Eg: oranges, mangoes etc) |  |  |  |  |  |  |
| Fresh fruit juice (squeezed from oranges, mangoes etc) |  |  |  |  |  |  |
| Vegetables (Eg: mchicha, spinach etc) |  |  |  |  |  |  |
| Milk eg: fresh or yoghurt (milk added to porridge, tea) |  |  |  |  |  |  |
| Chocolates, sweets |  |  |  |  |  |  |
| Sweetened beverages (Eg: soda, boxed juice, etc) |  |  |  |  |  |  |
| Cakes, biscuits |  |  |  |  |  |  |
| Doughnuts, mandazi |  |  |  |  |  |  |
| Snacks sold within or outside school premises by local street vendors (Eg: samosas, kababs, kachori, fried cassava, plantain, chips) |  |  |  |  |  |  |
| Meat |  |  |  |  |  |  |
| Poultry |  |  |  |  |  |  |
| Fish |  |  |  |  |  |  |
| Locally made ice cream (barafu, lambalamba) |  |  |  |  |  |  |
| Ice cream (industrial made eg: Azam, diaryland |  |  |  |  |  |  |
| Fast foods from restaurants (Eg: pizza, sausages, budger, chicken chips etc) |  |  |  |  |  |  |

**Section 2b**

This section ask about foods and drinks which are often available for home use, please select all that apply

- Fast foods bought from restaurants eg: chicken chips, samosas, kababs, sausages etc
- Fresh squeezed juice from fruits (home made)
- Sugary drinks such as sodas, artificial/ boxed juices (Azam, Fresh, Ceres, Juice cola etc)
- Chocolates, sweets
- Cakes, biscuits
- Doughnuts, mandazi, chapati
- Vegetables
- Fruits
- Milk and milk products (soured milk, fresh milk, yoghurt)

Please indicate whether the following items are available in your home for you to use, select all that apply

- Television
- Computer
- Cell phone
- Electronic gadgets (ipad, tablet etc)
- Video games (PlayStation, Nintendo etc)

Think about the local area around your home; within 10 – 15 minutes walk from home. Do you have any of the following in a walking distance from home? Please select all that apply

- Street vendors who sell snacks such as bajia, samosas, chips mayai
- Markets or local sellers who sell fruits and vegetables

The following questions ask about your neighborhood, please check all statements about your neighborhood

- There are playgrounds (public or private) eg: football pitch which can be used at any time
- There is much traffic which makes unpleasant to walk, or play around

Anthropometry measurements

Study ID:

| **Measure** | 1^st^ reading | 2^nd^ reading |
| --- | --- | --- |
| Height (cm) |  |  |
| Weight (kg) |  |  |
| Waist circumference (inches/ cm) |  |  |
| Hip circumference (inches/ cm) |  |  |
| Mid upper arm circumference (MUAC) (inches/ cm) |  |  |
| Triceps (mm) |  |  |
| Subscapular thickness (mm) |  |  |
| Body fat % |  |  |
