# Supplementary material for: Prevalence and correlates of overweight and obesity among primary school children in Kilimanjaro, Tanzania
Source: PLoS One. 2021 Apr 22;16(4):e0249595. doi: 10.1371/journal.pone.0249595 (PMC8061999; doi:10.1371/journal.pone.0249595)
Supplement: S2 File — (DOC) [file pone.0249595.s003.doc]

**2019 Utafiti wa Mazoezi kwa vitendo na Ulaji**

**Namba ya utambulisho:_______________ Tarehe** ____ / ____ /

**dd mm yyyy**

**Jina la shule: _______________________ Umiliki: ___________________**

**Wilaya:______________________ Kata:_________________**

**Mchukua dodoso: _______________________**

Tafadhali soma kila swali kwa umakini, na ufikirie ni jibu gani linakujia akilini kwako kwanza. Chagua kisanduku kinachoendana zaidi na jibu lako, na uweke alama ya vema (✔).

Kumbuka: Huu sio mtihani kwahiyo hakuna jibu sahihi au lisilosahihi, ila unaombwa kuwa mkweli kujibu maswali utakayoulizwa. Ni muhimu kujibu maswali yote na majibu yako yaonekane kwa usahihi.

Usimuoneshe mtu majibu yako. Hakuna mtu yeyote anaekufahamu atakaeangalia dodoso hii baada ya kujaza.

**Socio demographic characteristics/ Tabia za kijamii**

Tarehe ya kuzaliwa _____/_____/_____ Umri (miaka)__________

dd mm yyyy

Jinsia

- Mvulana
- Msichana

Unasoma darasa la ngapi?

- Darasa la nne
- Darasa la tano
- Darasa la sita
- Darasa la saba

**Kipengele 1a**

Kwa maswali ya ukurasa huu, tafadhali tunaomba utuambie shughuli ulizofanya juma lililopita/ wiki iliyopita

**Shughuli zisizohitaji kutumia nguvu**

Maswali mengi yanakutaka ufikirie tu kuhusu SIKU 7 ZILIZOPITA, ila maswali machache yanauliza kuhusu shughuli unayofanyaga (katika wiki ya kawaida).

HAKUNA MAJIBU SAHIHI WALA YASIYO SAHIHI, HIVYO UNAOMBWA KUWA MKWELI KATIKA KUJIBU MASWALI HAYA.

UFAFANUZI: VITENDO VISIVYOTUMIA NGUVU ni vitendo kama kuangalia luninga, kucheza gemu za video, michezo ya kompyuta au kutumia intaneti. Hii inajumiusha muda uliotumia kuchezea simu, kuongea na simu au kutuma meseji na muda uliokaa bila kufanya shughuli yoyote.

Siku za shule **(Jumatatu – Ijumaa)**

1. a) Je kwa juma liliyopita, ni siku ngapi uliangalia luninga?

- Sikuangalia
- Siku 1
- Siku 2
- Siku 3
- Siku 4
- Siku 5

1. Ni kwa masaa ngapi uliangalia luninga?

- Sikuangalia kabisa
- Chini ya saa moja (dakika 1 hadi 30)
- Lisaa limoja (dakika 31 hadi 60)
- Lisaa limoja hadi mawili
- Zaidi ya masaa mawili

1. a) Je kwa juma lililopita, ni siku ngapi ulicheza michezo ya luninga na kompyuta au kutumia kompyuta kwa shughuli zisizo za shule?

- Sikufanya hivi
- Siku 1
- Siku 2
- Siku 3
- Siku 4
- Siku 5

1. Ni kwa masaa mangapi ulicheza michezo ya luninga na kompyuta au ulitumia kompyuta kwa shughuli zisizo za shule?

- Sikufanya hivi
- Chini ya saa moja (dakika 1 hadi 30)
- Kwa saa moja (dakika 31 hadi 60)
- Saa moja hadi mawili
- Zaidi ya masaa mawili

1. a) Ni kwa siku ngapi ulikaa kimya na kujisomea au kupumzika/kukaa na familia au marafiki bila kufanya kazi yoyote?

- Sikufanya hivi
- Siku 1
- Siku 2
- Siku 3
- Siku 4
- Siku 5

1. Ulitumia masaa mangapi kukaa kimya na kujisomea au kupumzika/kukaa na familia au marafiki bila kufanya kazi yoyote?

- Sikufanya hivi
- Chini ya saa moja (dakika 1 hadi 30)
- Kwa saa moja (dakika 31 hadi 60)
- Saa moja hadi mawili
- Zaidi ya masaa mawili

Siku za mwisho wa juma **(Jumamosi na Jumapili)**

1. Ulitazama luninga kwa masaa mangapi?

- Sikutazama kabisa
- Chini ya saa moja (dakika 1 hadi 30)
- Saa moja (dakika 31 hadi 60)
- Saa moja hadi mawili
- Zaidi ya masaa mawili

1. Ni kwa masaa mangapi ulicheza michezo ya luninga na kompyuta au ulitumia kompyuta kwa shughuli zisizo za shule?

- Sikufanya hivi
- Chini ya saa moja (dakika 1 hadi 30)
- Kwa saa moja (dakika 31 hadi 60)
- Saa moja hadi mawili
- Zaidi ya masaa mawili

1. Ulitumia masaa mangapi kusoma au kupumzika/kukaa bila kazi yoyote peke yako au na familia?

- Sikufanya vitu hivi
- Chini ya saa moja (dakika 1 hadi 30)
- Saa limoja (dakika 31 hadi 60)
- Saa moja hadi mawili
- Zaidi ya masaa mawili

**Mazoezi ya viungo na michezo**

Sehemu hii inauliza kuhusu mazoezi na michezo yaani, michezo aina zote, gemu, mazoezi na kazi za nyumbani au shuleni (wakati au baada ya masomo), jinsi unavyofika shuleni na mazoezi ambayo yanakufanya utumie nguvu na kupumua kwa haraka (mfano: kutembea au kuendesha baiskeli kwenda shule).

1. Katika wiki ya kawaida, ni kwa siku ngapi umeshiriki katika somo la mazoezi kwa vitendo (Hii inamaanisha vipindi vya kutoka nje na kucheza uwanjani - PE)

- Sikushiriki
- Kwa siku 1
- Kwa siku 2
- Kwa siku 3
- Kwa siku 4
- Kwa siku 5

1. Katika wiki ya kawaida, je ni kawaida yako kutembea kwenda shuleni/ ili kufika shuleni?

- NDIYO
- HAPANA

KAMA JIBU NI HAPANA KWA SWALI LA 8, RUKA SWALI LA 9 NA UENDELEE NA SWALI LA 10

1. Je huwa unatumia muda gani kutembea ili kufika shuleni?

- Chini ya dakika 5
- Dakika 5 mpaka 15
- Dakika 16 mpaka 30
- Dakika 31 mpaka saa 1
- Zaidi ya saa 1

1. Kwa siku za shule, unapoamka asubuhi ukiwa bado nyumbani ni kawaida yako kunafanya kazi za nyumbani kama vile kazi za bustani, kuteka maji, kufagia nk. kwa muda usiopungua dakika 10?

- NDIYO
- HAPANA

1. Kwa siku za shule, unaporudi nyumbani kutoka shuleni ni kawaida yako kufanya kazi za nyumbani kama vile kazi za bustani, kuteka maji, kufagia nk. kwa muda usiopungua dakika 10?

- NDIYO
- HAPANA

1. Wakati ukiwa shuleni, huwa unatumia muda wako wa mapuziko kufanya mazoezi/ kucheza michezo kama mpira wa kikapu, tikri, mpira wa miguu, kuruka kamba nk?

- NDIYO
- HAPANA

1. Je wazazi wako huwa wanakushawishi kufanya mazoezi au wanakushauri kwenda kwenda nje, au uwanjani kucheza?

- NDIYO
- HAPANA

**Kipengele cha 2a**

1. Ulaji wa chakula mara kwa mara

Kipengele hiki kinauliza kuhusu ulaji wa vyakula vifuatavyo mara kwa mara (Tafadhali weka alama ya vema (✔) kwenye kisanduku kimoja tu katika kila mstari kinachoelezea ulaji wako wa kawaida)

|  | Sijawahi | Mara moja kwa siku | Zaidi ya mara moja kwa siku | Mara 3 – 6 kwa wiki | Mara moja au mara mbili kwa wiki | Kila siku, zaidi ya mara moja |
| --- | --- | --- | --- | --- | --- | --- |
| Matunda (Mfano: machunga, maembe nk) |  |  |  |  |  |  |
| Juisi ya matunda (Mfano: itokanayo na machungwa, maembe, nk) |  |  |  |  |  |  |
| Mbogamboga (Mfano: mchicha, spinachi nk) |  |  |  |  |  |  |
| Maziwa freshi au mtindi (mfano: maziwa ya chai, au yalioongezwa kwenye uji) |  |  |  |  |  |  |
| Chokoleti, pipi |  |  |  |  |  |  |
| Vinywaji vyenye sukari (Mfano: soda, Juisi za dukani kama Azam,Ceres nk) |  |  |  |  |  |  |
| Keki, biskuti |  |  |  |  |  |  |
| Donati, mandazi |  |  |  |  |  |  |
| Hasusa/ vitafunio zinazouzwa mazingira ya shule/ nje ya shule na mama ntilie (Mfano: sambusa, kababu, kachori, mihogo ya kukaanga, ndizi za kukaanga, chipsi) |  |  |  |  |  |  |
| Nyama |  |  |  |  |  |  |
| Nyama ya kuku |  |  |  |  |  |  |
| Samaki |  |  |  |  |  |  |
| Barafu, lambalamba |  |  |  |  |  |  |
| Ice cream za dukani (Mfano: Azam, diaryland) |  |  |  |  |  |  |
| Vyakula vya kukaanga vya mahotelini (Eg: pizza, sausages, baga, chipsi kuku, chipsi mayai, soseji nk) |  |  |  |  |  |  |

**Sehemu ya 2b**

1. Kipengele hiki kinauliza kuhusu vyakula na vinywaji ambavyo kwa kawaida huwa vinapatikana kwa matumizi ya nyumbani. Tafadhali weka alama ya vema kuonyesha vyakula na vinywaji ambavyo kwa kawaida vinavyokuwapo nyumbani kwenu (✔).

- Vyakula vya kununua mahotelini au kwenye migahawa, mama ntilie mfano: sambusa, chipsi kuku, kababu, soseji nk
- Juisi freshi ya matunda iliyotengenezwa nyumbani
- Vinywaji vyenye sukari: mfano soda, juisi za maboxi (Azam, Fresh, Ceres, Juice cola etc)
- Chokoleti/ pipi
- Donati, mandazi
- Sambusa, kababu
- Mboga za majani
- Matunda
- Maziwa: Mfano (mtindi, maziwa fresh, yogati)

1. Tafadhali weka alama ya vema (✔) kuonyesha kama vifaa vifuatavyo huwa vinapatikana/ vipo nyumbani kwenu kwa ajili ya matumizi

- Televisheni/ Luninga
- Kompyuta
- Simu
- Vifaa vya kielektroniki (Mfano: ipad, tablet nk.)
- Gemu za video (Mfano: PlayStation, Nintendo nk.)

1. Sasa tunaomba ufikirie kuhusu mazingira ya karibu na nyumbani kwenu unayokoishi; kati ya umbali wa kutembea kwa dakika 10 hadi 15. Je vitu vilivyoorodheshwa hapo chini huwa vinapatikana katika umbali huyo? Weka alama ya vema (✔) kwa vyote vinayopatikana

- Mama ntilie, migahawa au magenge wanaouza vitafunio kama vile bajia, sambusa, chipsi mayai nk
- Soko au watembeza mikokoteni, au watembezaji kwa makapu wanaouza matunda na mbogamboga

1. Maswali yafuatayo yanauliza kuhusiana na ujirani, tafadhali weka lama ya vema (✔) kwa misemo yote ambayo inaelezea kuhusu maeneo ya jirani na unapoishi

- Kuna viwanja vya michezo (vya jumuiya, au vya kulipia) mfano: uwanja wa mpira ambao unaweza kutumika kwa wakati wowote unapohitaji kucheza
- Kuna magari mengi yanayosababisha ugumu katika kutembea kwa mguu, au kuwa huru kucheza

Anthropometry measurements

Utambulisho ID:

| **Measure** | 1^st^ reading | 2^nd^ reading |
| --- | --- | --- |
| Height (cm) |  |  |
| Weight (kg) |  |  |
| Waist circumference (inches/ cm) |  |  |
| Hip circumference (inches/ cm) |  |  |
| Mid upper arm circumference (MUAC) (inches/ cm) |  |  |
| Triceps (mm) |  |  |
| Subscapular thickness (mm) |  |  |
| Body fat % |  |  |
